# Behavioral deviations: healthcare-seeking behavior of chronic disease patients with intention to visit primary health care institutions

**DOI:** 10.1186/s12913-023-09528-y

**Published:** 2023-05-16

**Authors:** Shiyin Wu, Shanshan Du, Ruimei Feng, Wenbin Liu, Weimin Ye

**Affiliations:** 1 Department of Health Management, School of Health Management, Fujian Medical University, Fuzhou, Fujian China; 2 School of Public Health, Fujian Medical University, 1 Xuefubei Road, Minhou District, Fuzhou, 350122 China

**Keywords:** Behavior deviations, Chronic disease, Andersen’s behavioral model, Healthcare-seeking behavior

## Abstract

**Background:**

Although primary health care (PHC) has been proven to be effective in preventing and treating chronic diseases, the visits rate of PHC institutions is still not ideal. Some patients initially express a willingness to visit PHC institutions but end up seeking health services at non-PHC institutions, and the reasons for this behavior remain unclear. Therefore, the objective of this study is to analyze the factors that contribute to behavioral deviations among chronic disease patients who originally intended to visit PHC institutions.

**Methods:**

Data were collected from a cross-sectional survey among chronic disease patients with original intention to visit PHC institutions in Fuqing City, China. The analysis framework was guided by Andersen’s behavioral model. Logistic regression models were employed to analyze the factors affecting the behavioral deviations among chronic disease patients with a willingness to visit PHC institutions.

**Results:**

A total of 1,048 individuals were finally included and about 40% of the participants with the original willingness to seek care from PHC institutions finally chose non-PHC institutions in their subsequent visits. The results of logistic regression analyses indicated that at the predisposition factor level, older participants (aOR_60-69_ = 0.602, *P* < 0.01; aOR_70-75_ = 0.475, *P* < 0.01) were less likely to have behavioral deviations. At the enabling factor level, compared to those covered by Urban Employee Basic Medical Insurance (UEBMI) and not reimbursed, those covered by Urban–Rural Resident Basic Medical Insurance (URRBMI) (aOR = 0.297, *P* < 0.01), and those answering that reimbursement from medical institutions was convenient (aOR = 0.501, *P* < 0.01) or very convenient (aOR = 0.358, *P* < 0.001) were less likely to have behavioral deviations. At the need factor level, participants who visited PHC institutions due to illness last year (aOR = 0.348, *P* < 0.001) and with polypharmacy (aOR = 0.546, *P* < 0.01) were less likely to have behavioral deviations compared to those without the visit of PHC institutions and not taking polypharmacy, respectively.

**Conclusions:**

The deviations between the original willingness of PHC institution visits and subsequent behavior among chronic disease patients were associated with a number of predisposing, enabling, and need factors. Developing the health insurance system, strengthening the technical capacity of PHC institutions, and steadily developing a new concept of orderly healthcare-seeking behavior among chronic disease patients, will help promote their access to PHC institutions as well as improve the effectiveness of the tiered medical system for chronic disease care.

**Supplementary Information:**

The online version contains supplementary material available at 10.1186/s12913-023-09528-y.

## Background

Due to their high prevalence and associated mortality, chronic diseases have posed great threats to human health [1]. Especially in the context of increasing life expectancy and aging, multimorbidity, namely patients with two or more chronic diseases, is also becoming increasingly common [2, 3]. In China, among people with chronic diseases, the overall prevalence of a single chronic disease is 38.1%, while the prevalence of multimorbidity is 61.9% [4]. Since chronic diseases lead to the imbalance of various physiological functions of patients, aggravate health damage, and impose growing burdens on individuals, families, society, and healthcare systems, it is urgent to reverse this serious situation [5, 6].

Primary health care (PHC) is one of the essential strategies to combat chronic diseases, which refers to the first contact, longitudinal, comprehensive and coordinated care provided to individuals [7–9]. It has been demonstrated that effective primary care is associated with enhanced access to healthcare services, better health outcomes, increased health equity, cost effectiveness, and early management of health problems [10–12]. In China’s current tiered medical system, PHC institutions, which are the providers of PHC, not only provide general disease diagnosis and treatment but also provide essential public health services [13, 14]. However, in China, many chronic disease patients tend to visit secondary and tertiary hospitals rather than PHC institutions for their basic medical services due to various considerations. Bypassing PHC institutions to seek care from secondary and tertiary healthcare facilities will aggravate the patients’ economic burden, bring about tremendous waste in healthcare resources, and ultimately affect the performance of the health system [15]. Thus, it is of great significance to investigate the influencing factors of chronic disease patients’ reluctance to see a doctor at PHC institutions, especially those who were initially willing to do so.

Previous studies on factors influencing patients’ healthcare-seeking behaviors have paid attention to the individuals [16–19], providers [20–23], and social dimensions [24–26]. These studies have proved that individuals’ characteristics, quality of medical services, health care accessibility, social culture, and their experiences with medical treatment were the key factors impacting the patient’s medical treatment seeking behavior. For instance, a study in Burkina Faso showed that social communication about the disease could effectively increase the appropriate healthcare-seeking behavior rate among patients [27]. Besides, researchers also investigated the impact of disease factors on healthcare-seeking behavior. A nationwide longitudinal study in China found that patients with multimorbidity made more use of PHC and hospitalization services compared to those with just one chronic condition [28]. Similarly, another study in 16 European countries indicated that there was a strong link between an increasing number of chronic diseases and more healthcare utilization [29], which was consistent with studies conducted in both developed and developing countries [30–32]. But there are still several areas that the previous studies could be improved. First, researches on this topic mainly focus on the group of the elderly [26, 33, 34], rural residents [35], or patients with specific diseases [4, 17, 19], etc. Few studies have focused on the group willing to visit PHC institutions, while implementing interventions for such a group is more effective in increasing PHC institutions’ attendance. Second, the influencing factors of patients’ healthcare-seeking behavior are multiple. However, many studies only analyzed the effect of specific factors such as medical security and social support [25, 36], without integrating the impact of other factors, which may lead to difficulties in developing effective interventions. Third, although some scholars have studied chronic disease patients’ healthcare-seeking behavior, there is little research comparing the influencing factors of differences in healthcare-seeking behavior between patients with a single chronic condition and those with multimorbidity.

Therefore, this study will take chronic disease patients who are willing to visit PHC institutions as the research subjects and use corresponding theoretical model as the analysis framework. It aims to analyze the determinants of chronic disease patients’ behavioral deviations from their original willingness to seek health services at PHC institutions, and to compare differences in healthcare-seeking behavior between patients with a single chronic condition and those with multimorbidity. The findings will not only reveal reasons for the behavioral deviations among chronic disease patients willing to visit PHC institutions, but also provide a basis for developing effective interventions to guide patients’ healthcare-seeking behavior.

## Methods

### Study design

The cross-sectional study was conducted as part of the Fuqing Cohort Study. Fuqing Cohort Study is a large prospective study recruiting all residents aged 35–75 years in Fuqing City, which provides physical examinations, biospecimen collection and questionnaires for residents, as well as long-term and continuous follow-up observations. Between Jan 2019 and June 2021, Fuqing Cohort Study was conducted in Gaoshan Town of Fuqing City, a coastal town in southeastern China with 23 administrative villages and two communities. A total of 25,930 residents aged 35–75 years in Gaoshan Town were invited to participate in the cohort study, and 10,262 participants completed the survey with a response rate of 39.6%.

### Data source and participants

The data of this study were from the Fuqing Cohort questionnaire survey between June 2020 and June 2021, and the extracted items included healthcare expenditure in the previous year, medical insurance, PHC visits due to illness, and so on (see Additional file 1). Thus, a total of 7,074 data were originally included.

Since our study focused on investigating the behavioral deviations among chronic disease patients with a prior intention to visit PHC institutions, the inclusion criteria for respondents were (1) individuals who were willing to visit PHC institutions, (2) individuals diagnosed with non-communicable diseases by medical institutions or physicians, including diabetes, hypertension, stroke, hyperuricemia, cancer, hyperthyroidism, hypothyroidism, thyroid nodules, liver disease, and coronary heart disease. The exclusion criteria for respondents were (1) individuals who were unwilling to visit PHC institutions and (2) individuals without non-communicable diseases. Furthermore, as the study aimed to compare differences in healthcare-seeking behavior between patients with a single chronic condition and those with multimorbidity, we divided study subjects based on their chronic disease status (i.e., single morbidity, or multimorbidity). After processing the data and removing those participants with missing values in dependent variables (For details see Additional file 2), the final sample included 1,048 respondents, which could fully meet the basic requirement that the sample size be set at least five times the survey question items [37].

The study has been approved by the Ethics Review Committee of Fujian Medical University (approval number, [2017-07] and [2020-58]). All participants in Fuqing Cohort provided written informed consent.

### Measures

#### Outcome variable: behavioral deviations

As the study focused on investigating behavioral deviations among chronic disease patients with original intention to visit PHC institutions, we defined the behavioral deviations as chronic disease patients who were willing to obtain care from PHC institutions actually seeking health services at non-PHC institutions. And this dependent variable was evaluated by the following questions, “Which healthcare provider did you prefer to visit when you had common or frequently-occurring diseases?” and “In the past year, which type of medical institutions did you visit when you had common or frequently-occurring diseases?” Respondents chose their answers from the following settings, PHC institutions (including village clinics, community healthcare centers, and township hospitals) and non-PHC institutions (including general hospitals, Chinese medicine hospitals, and specialized hospitals). According to their answers, we classified the dependent variable into two categories: 0 = no behavioral deviations (chronic disease patients who are willing to visit PHC institutions actually seek care from PHC institutions), and 1 = behavioral deviations (chronic disease patients who are willing to visit PHC institutions actually seek care from non-PHC institutions). Table 1 presents the definitions of variables.

**Table1:** Definition of variables included in the study

Variables	Description
**Dependent variable**	
Behavioral deviations	1 = if a patient who is willing to obtain care from primary health care (PHC) institutions actually seeks health services at non-PHC institutions; 0 = otherwise
**Independent variable**	
Sex	1 = male; 2 = female
Age	1 = 35 ~ 59 years; 2 = 60 ~ 69 years; 3 = 70 ~ 75 years
Education	1 = illiteracy; 2 = elementary school; 3 = middle school; 4 = high school or higher
Marital status	1 = married; 0 = others (single, divorced, widowed and separated)
Healthcare expenditure in the previous year	1 = 0 ~ 3,000 CNY; 2 = 3,000 ~ 4,999 CNY; 3 = 5,000 ~ 9,999 CNY; 4 = 10,000 CNY or more
Medical insurance	1 = Urban Employee Basic Medical Insurance (UEBMI); 2 = Urban and Rural Residents Basic Medical Insurance (URRBMI)
Convenience of medical cost reimbursement	1 = didn’t apply for medical reimbursement; 2 = inconvenient; 3 = convenient; 4 = very convenient
PHC visits due to illness	1 = yes (visit PHC institutions due to illness in the past year); 0 = no
Polypharmacy	1 = yes (take two or more classes of drugs); 0 = no
Need of guidance on health issues	1 = yes; 0 = no

#### Independent variables

**Fig. 1:**
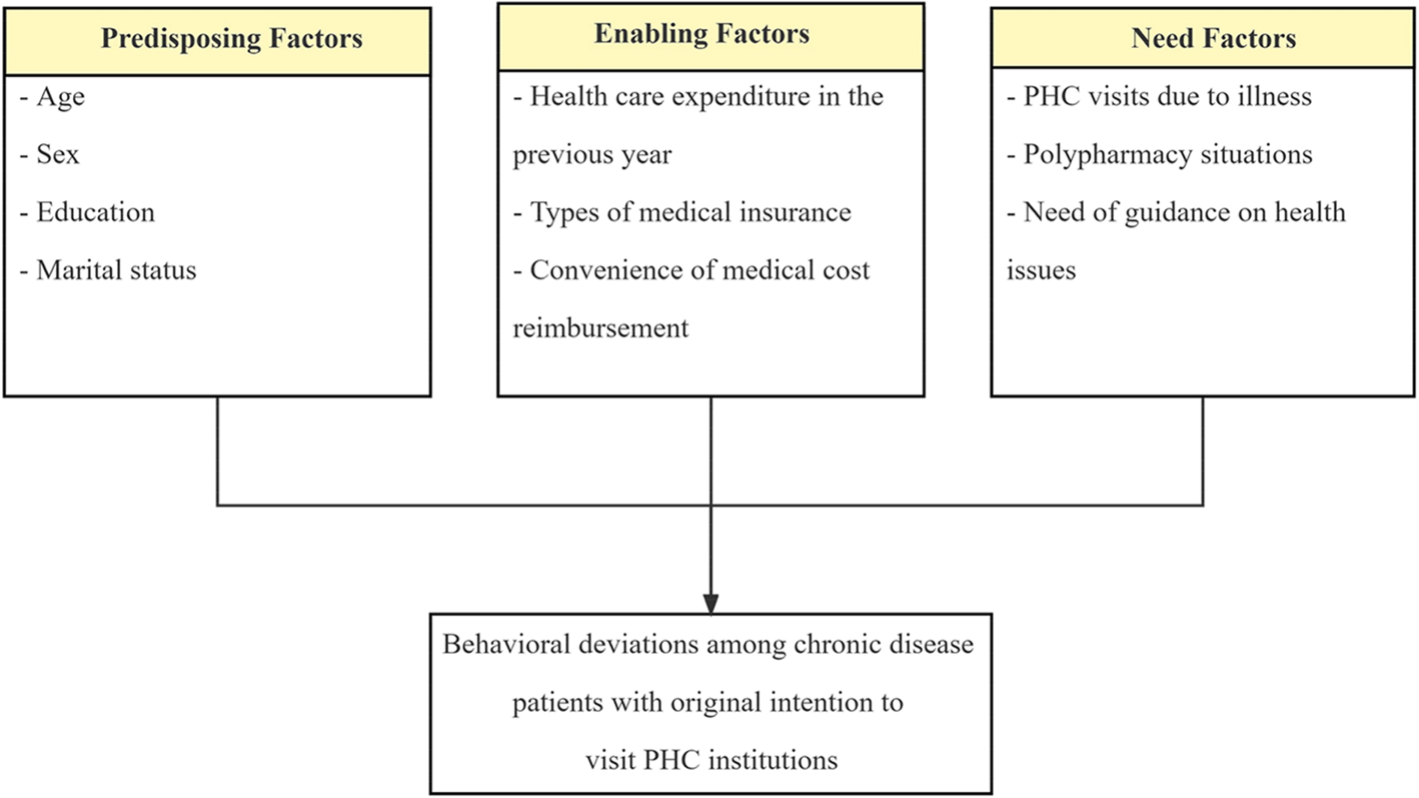
Theoretical model and variables

This study took the original version of Andersen’s behavioral model [38] to guide our analysis of behavioral deviation determinants among chronic disease patients. The Andersen Model is the most classical and influential theoretical framework being applied in various fields, such as public health, medicine, and sociology. In particular, it has been used to examine healthcare service utilization in providing a basis for systematic investigations of factors impacting healthcare-seeking behavior. Thus, we hypothesize that chronic disease patients’ behavioral deviations from their original willingness of seeking health services at PHC institutions are based on three factors, including predisposing, enabling, and need factors. And the independent variables were selected and grouped for three criteria: confirmed associations with patients’ healthcare-seeking behavior [16, 19, 28, 29, 39, 40]; availability in the Fuqing Cohort dataset; and accordance with the theoretical model as follows (Fig. 1).

First, predisposing factors represent the personal characteristics that tend to utilize health services [41]. The predisposing factors in this paper include age, sex, education, and marital status.

Second, enabling factors ensure the possibility of access to health care, namely the financial and social factors that affect people’s healthcare-seeking behavior [41]. The enabling variables in this study include healthcare expenditure in the previous year, types of medical insurance, and convenience of medical cost reimbursement.

Third, need factors represent people’s perceptions of their general health status, functional status, and how the severity of their disease affects their healthcare-seeking behavior [42]. The need factors in this study include PHC visits due to illness, polypharmacy situations, and need of guidance on health issues. The PHC visits due to illness was defined as whether the patients visited PHC institutions due to illness in the past year, which may influence their judgment of their own health status, and subsequently affected their healthcare-seeking behavior. Polypharmacy situations referred to whether the patients took two or more classes of drugs, which to some extent reflected the severity of patient’s disease and could influence their healthcare-seeking behavior. Need of guidance on health issues meant that patients decided whether they needed professional health advice based on their general health status.

### Statistical analysis

First, descriptive statistics were used to summarize the characteristics of the overall chronic disease patient population, single chronic disease population, and multiple chronic disease population. Second, Chi-squared tests were used to examine the differences in actual healthcare-seeking behavior across groups. Third, to analyze the determinants of the deviations between actual health services seeking behavior and their original willingness of PHC institutions visit, the logistic regression model was employed, in total, and among the group of patients with single or multiple chronic diseases, respectively. In the multivariate logistic model, whether individuals had behavioral deviations or not was set as the dependent variable, while the predisposing factors (sex, age, education, and marital status), enabling factors (healthcare expenditure in the previous year, types of medical insurance, and convenience of medical cost reimbursement), and need factors (PHC visits due to illness, polypharmacy situations, and need of guidance on health issues) were set as independent variables. In addition, to mitigate potential bias, the missing values were substituted by the mean value. The analysis was repeated with the substituted data and no significant differences were found between the two analyses. The final multivariate analysis was based on the substituted data. The results were presented as the unadjusted Odds Ratio (uOR) and adjusted Odds Ratio (aOR) with 95% confidence intervals (CI) obtained from univariate and multivariate logistic models, respectively. Finally, robustness tests were conducted by replacing the explanatory variables and supplementary variables to further test the results obtained. STATA version 16 and PASS version 15 were applied to conduct data analysis.

## Results

### Demographic characteristics of the sample

Table 2 shows the demographic characteristics of the survey participants. The analytical sample included 1,048 respondents, among whom 404 (38.55%) reported having multiple chronic diseases. In addition, since some respondents refused or failed to provide exact answers to certain questions, there were missing data for the following variables: education, marital status, the convenience of medical cost reimbursement, and PHC visits due to illness. As shown in Table 2, more than half of the participants were female (68.32%). Respondents with the age of fewer than 60 years, 60–69 years, and 70–75 years accounted for 47.14%, 37.88%, and 14.98% of the total sample size, respectively. Only 7.25% of the participants received senior high school education or higher levels and more than four-fifths of respondents (89.22%) were married. About 63.36% of the individuals reported their healthcare expenditures in the past year were less than 3,000 Chinese Yuan (CNY), 11.16% between 3,000 and 4,999 CNY, 12.50% between 5,000 and 9,999 CNY, and 12.98% greater than 10,000 CNY, respectively. Urban–Rural Resident Basic Medical Insurance (URRBMI) programs covered 97.14% of respondents. Over half of the chronic disease patients (55.06%) considered medical cost reimbursement very convenient. More than two-thirds of individuals (69.85%) visited primary care facilities due to illness in the previous year, and about 26.53% of the study sample took multiple classes of medications. In terms of needing guidance on health issues, 68.89% needed guidance related to their own health issues.

**Table 2:** Characteristics of 1048 respondents

**Variables**	**Total**	**Single chronic disease**	**Multimorbidity**
	**Frequency (%)**	**Frequency (%)**	**Frequency (%)**
**Predisposing factors**			
Sex			
Male	332 (31.68)	201 (31.21)	131 (32.43)
Female	716 (68.32)	443 (68.79)	273 (67.57)
Age			
35–59	494 (47.14)	336 (52.17)	158 (39.11)
60–69	397 (37.88)	222 (34.47)	175 (43.32)
70–75	157 (14.98)	86 (13.35)	71 (17.57)
Education			
Illiteracy	384 (36.64)	220 (34.16)	164 (40.59)
Elementary school	370 (35.31)	233 (36.18)	137 (33.91)
Middle school	212 (20.23)	134 (20.81)	78 (19.31)
High school or higher	76 (7.25)	53 (8.23)	23 (5.69)
Missing	6 (0.57)	4 (0.62)	2 (0.50)
Marital status			
Others (single, divorced, widowed and separated)	107 (10.21)	55 (8.54)	52 (12.87)
Married	935 (89.22)	585 (90.84)	350 (86.63)
Missing	6 (0.57)	4 (0.62)	2 (0.50)
**Enabling factors**			
Healthcare expenditure in the previous year(CNY)			
< 3,000	664 (63.36)	452 (70.19)	212 (52.48)
3,000–4,999	117 (11.16)	69 (10.71)	48 (11.88)
5,000–9,999	131 (12.50)	72 (11.18)	59 (14.60)
> = 10,000	136 (12.98)	51 (7.92)	85 (21.04)
Medical insurance			
UEBMI	30 (2.86)	17 (2.64)	13 (3.22)
URRBMI	1018 (97.14)	627 (97.36)	391 (96.78)
Convenience of medical cost reimbursement			
No reimbursed	133 (12.69)	89 (13.82)	44 (10.89)
Inconvenient	96 (9.16)	50 (7.76)	46 (11.39)
Convenient	226 (21.56)	156 (24.22)	70 (17.33)
Very convenient	577 (55.06)	339 (52.64)	238 (58.91)
Missing	16 (1.53)	10 (1.55)	6 (1.49)
**Need factors**			
PHC visits due to illness			
No	315 (30.06)	200 (31.06)	115 (28.47)
Yes	732 (69.85)	443 (68.79)	289 (71.53)
Missing	1 (0.10)	1 (0.16)	
Polypharmacy			
No	770 (73.47)	553 (85.87)	217 (53.71)
Yes	278 (26.53)	91 (14.13)	187 (46.29)
Need of guidance on health issues			
No	326 (31.11)	196 (30.43)	130 (32.18)
Yes	722 (68.89)	448 (69.57)	274 (67.82)

### Behavioral deviations among chronic disease patients with intention to visit PHC institutions

Table 3 presents the behavior deviations of chronic disease patients who were willing to visit PHC institutions. In the chronic disease population, among predisposing factors, sex, age, and education were significantly associated with behavioral deviations among patients with intention to visit PHC institutions (*P* < 0.05). Among enabling factors, healthcare expenditures in the past year, medical insurance, and convenience of medical cost reimbursement all significantly correlated with behavioral deviations (*P* < 0.05). Among the need factors, PHC visits due to illness in the previous year was significantly linked to behavioral deviations (*P* < 0.001).

**Table 3:** Behavior deviations of chronic disease patients who are willing to visit primary health care institutions^a^

Variables	Total				Single chronic disease				Multimorbidity			
	No Deviation (%)	Deviation (%)	*χ*^*2*^	*P*-value	No Deviation (%)	Deviation (%)	*χ*^*2*^	*P*-value	No Deviation (%)	Deviation (%)	*χ*^*2*^	*P*-value
**Predisposing factors**												
Sex			5.110	0.024			1.028	0.311			5.483	0.019
Male	180(54.22)	152(45.78)			114(56.72)	87(43.28)			66(50.38)	65(49.62)		
Female	441(61.59)	275(38.41)			270(60.95)	173(39.05)			171(62.64)	102(37.36)		
Age			12.833	0.002			4.606	0.100			11.374	0.003
35–59	266(53.85)	228(46.15)			187(55.65)	149(44.35)			79(50.00)	79(50.00)		
60–69	248(62.47)	149(37.53)			142(63.96)	80(36.04)			106(60.57)	69(39.43)		
70–75	107(68.15)	50(31.85)			55(63.95)	31(36.05)			52(73.24)	19(26.76)		
Education			12.179	0.007			3.901	0.272			16.755	0.001
Illiteracy	250(65.10)	134(34.90)			136(61.82)	84(38.18)			114(69.51)	50(30.49)		
Elementary school	220(58.51)	156(41.49)			142(59.92)	95(40.08)			78(56.12)	61(43.88)		
Middle school	115(54.25)	97(45.75)			81(60.45)	53(39.55)			34(43.59)	44(56.41)		
High school or higher	36(47.37)	40(52.63)			25(47.17)	28(52.83)			11(47.83)	12(52.17)		
Marital status			0.499	0.480			0.266	0.606			0.206	0.650
Others (single,divorced,widowed and separated)	60(56.07)	47(43.93)			31(56.36)	24(43.64)			29(55.77)	23(44.23)		
Married	561(59.62)	380(40.38)			353(59.93)	236(40.07)			208(59.09)	144(40.91)		
**Enabling factors**												
Healthcare expenditure in the previous year			143.103	< 0.001			99.264	< 0.001			57.830	< 0.001
< 3,000	477(71.84)	187(28.16)			324(71.68)	128(28.32)			153(72.17)	59(27.83)		
3,000–4,999	61(52.14)	56(47.86)			28(40.58)	41(59.42)			33(68.75)	15(31.25)		
5,000–9,999	53(40.46)	78(59.54)			24(33.33)	48(66.67)			29(49.15)	30(50.85)		
> = 10,000	30(22.06)	106(77.94)			8(15.69)	43(84.31)			22(25.88)	63(74.12)		
Medical insurance			8.596	0.003			6.622	0.010			2.261	0.133
UEBMI	10(33.33)	20(66.67)			5(29.41)	12(70.59)			5(38.46)	8(61.54)		
URRBMI	611(60.02)	407(39.98)			379(60.45)	248(39.55)			232(59.34)	159(40.66)		
Convenience of medical cost reimbursement			19.550	< 0.001			13.361	0.004			8.978	0.030
No reimbursed	61(45.86)	72(54.14)			43(48.31)	46(51.69)			18(40.91)	26(59.09)		
Inconvenient	49(51.04)	47(48.96)			26(52.00)	24(48.00)			23(50.00)	23(50.00)		
Convenient	129(57.08)	97(42.92)			85(54.49)	71(45.51)			44(62.86)	26(37.14)		
Very convenient	382(64.42)	211(35.58)			230(65.90)	119(34.10)			152(62.30)	92(37.70)		
**Need factors**												
PHC visits due to illness			34.206	< 0.001			17.723	< 0.001			17.088	< 0.001
No	144(45.71)	171(54.29)			95(47.50)	105(52.50)			49(42.61)	66(57.39)		
Yes	477(65.08)	256(34.92)			289(65.09)	155(34.91)			188(65.05)	101(34.95)		
Polypharmacy			3.052	0.081			0.399	0.528			4.355	0.037
No	444(57.66)	326(42.34)			327(59.13)	226(40.87)			117(53.92)	100(46.08)		
Yes	177(63.67)	101(36.33)			57(62.64)	34(37.36)			120(64.17)	67(35.83)		
Need of guidance on health issues			0.025	0.873			0.138	0.710			0.498	0.480
No	192(58.90)	134(41.10)			119(60.71)	77(39.29)			73(56.15)	57(43.85)		
Yes	429(59.42)	293(40.58)			265(59.15)	183(40.85)			164(59.85)	110(40.15)		

^a^Data analysis was based on the substituted data

Among the respondents who had a single chronic disease, for enabling factors, the patients with different healthcare expenditures in the past year, medical insurance, and convenience of medical cost reimbursement were all significantly different in behavioral deviations from the original willingness to visit PHC institutions (*P* < 0.05). For the need factors, patients with different PHC visits due to illness in the previous year were significantly different in behavioral deviations from the intention to visit PHC institutions (*P* < 0.001).

Among the respondents with multiple chronic diseases, for the level of predisposing factors, sex, age, and education were significantly correlated with the behavioral deviations (*P* < 0.05). For enabling factors, patients with different healthcare expenditures in the past year and the convenience of medical cost reimbursement were significantly different in the behavioral deviations (*P* < 0.05). For the need factors, the patients with different PHC visits due to illness in the previous year and polypharmacy situations were significantly different in the behavioral deviations (*P* < 0.05).

### Determinants of behavioral deviations from the original intention among respondents who were willing to visit PHC institutions

In this study, the statistical power was 80.47% and the multiple collinearity test showed that the VIF of each factor was less than 5, indicating that there were no multicollinearity noted among the analyzed factors (Specific data is shown in Additional file 3). In addition, we also conducted robustness tests by replacing the explanatory variables and supplementary variables, and the results strongly verified the robustness and reliability of the regression results (Specific data is shown in Additional file 4). Table 4 presents the results of logistic regression analysis by mutually adjusting variables listed in the table. The results demonstrated that age, medical care expenditure in the previous year, type of medical insurance, convenience of medical cost reimbursement, PHC visits due to illness last year, and polypharmacy situations had significant impacts on behavioral deviations among patients with chronic disease.

**Table 4:** Logistic regression analysis on the factors influencing behavior deviations^a^

Variables	Total^b^		Single chronic disease^c^		Multimorbidity^d^	
	*uOR*(95%*CI*)	*aOR*(95%*CI*)	*uOR*(95%*CI*)	*aOR*(95%*CI*)	*uOR*(95%*CI*)	*aOR*(95%*CI*)
**Predisposing factors**						
Sex (Ref = Male)						
Female	0.74(0.57–0.96)*	0.87(0.60–1.26)	0.84(0.60–1.18)	1.03(0.64–1.66)	0.61(0.40–0.92)*	0.72(0.38–1.36)
Age (Ref = 35–59)						
60–69	0.70(0.54–0.92)*	0.60(0.43–0.85)**	0.71(0.50–1.00)	0.61(0.39–0.96)*	0.65(0.42–1.00)	0.61(0.35–1.06)
70–75	0.55(0.37–0.80)**	0.48(0.30–0.76)**	0.71(0.43–1.16)	0.56(0.30–1.05)	0.36(0.20–0.67)**	0.34(0.16–0.73)**
Education (Ref = Illiteracy)						
Elementary school	1.32(0.99–1.77)	1.11(0.77–1.60)	1.08(0.74–1.58)	0.96(0.60–1.53)	1.78(1.11–2.86)*	1.43(0.78–2.61)
Middle school	1.57(1.12–2.22)**	1.07(0.68–1.69)	1.06(0.68–1.65)	0.78(0.44–1.38)	2.95(1.69–5.15)***	1.98(0.88–4.45)
High school or higher	2.07(1.26–3.41)**	1.30(0.68–2.47)	1.81(0.99–3.32)	1.28(0.59–2.80)	2.49(1.03–6.02)*	1.37(0.41–4.55)
Marital status (Ref = Others)						
Married	0.87(0.58–1.29)	0.66(0.41–1.06)	0.86(0.49–1.50)	0.63(0.32–1.22)	0.87(0.49–1.57)	0.69(0.34–1.44)
**Enabling factors**						
Healthcare expenditure in the previous year (Ref = < 3,000)						
3,000–4,999	2.34 (1.57–3.49)***	3.43(2.21–5.34)***	3.71(2.20–6.25)***	4.89(2.78–8.61)***	1.18(0.60–2.33)	1.89(0.88–4.07)
5,000–9,999	3.75(2.55–5.53)***	5.71(3.69–8.84)***	5.06(2.98–8.61)***	6.84(3.84–12.17)***	2.68(1.48–4.85)**	4.20(2.08–8.50)***
> = 10,000	9.01(5.81–13.98)***	15.60(9.50–25.64)***	13.61(6.23–29.74)***	19.02(8.23–43.99)***	7.43(4.20–13.14)***	14.19(7.14–28.22)***
Medical insurance (Ref = UEBMI)						
URRBMI	0.33(0.15–0.72)**	0.30(0.13–0.70)**	0.27(0.10–0.78)*	0.24(0.08–0.78)*	0.43(0.14–1.33)	0.40(0.10–1.52)
Convenience of medical cost reimbursement (Ref = No reimbursed)						
Inconvenient	0.81(0.48–1.38)	0.68(0.37–1.25)	0.86(0.43–1.73)	0.70(0.31–1.55)	0.69(0.30–1.59)	0.66(0.24–1.78)
Convenient	0.64(0.41–0.98)*	0.50(0.31–0.82)**	0.78(0.46–1.32)	0.63(0.35–1.13)	0.41(0.19–0.89)*	0.27(0.11–0.70)**
Very convenient	0.47(0.32–0.68)***	0.36(0.23–0.55)***	0.48(0.30–0.78)**	0.38(0.22–0.65)***	0.42(0.22–0.81)**	0.28(0.13–0.62)**
**Need factors**						
PHC visits due to illness (Ref = No)						
Yes	0.45(0.35–0.59)***	0.35(0.26–0.48)***	0.49(0.35–0.68)***	0.39(0.26–0.57)***	0.40(0.26–0.62)***	0.28(0.16–0.48)***
Polypharmacy (Ref = No)						
Yes	0.78(0.59–1.03)	0.55(0.38–0.78)**	0.86(0.55–1.36)	0.65(0.37–1.14)	0.65(0.44–0.98)*	0.58(0.34–0.97)*
Need of guidance on health issues (Ref = No)						
Yes	0.87(0.75–1.28)	0.85(0.62–1.15)	1.07(0.76–1.50)	0.90(0.60–1.34)	0.86(0.56–1.31)	0.73(0.43–1.21)
**Constant**	-	2.514	-	2.476	-	2.709

*uOR* Unadjusted odds ratio, *aOR* Adjusted odds ratios

^*^P < 0.05

^**^P < 0.01

^***^P < 0.001

^a^Variables listed in the table were mutually adjusted

^b^The coefficient of determination (R^2^) for this model was 0.190

^c^The coefficient of determination (R^2^) for this model was 0.185

^d^The coefficient of determination (R^2^) for this model was 0.237

Among the group of patients with a single chronic disease, for predisposing factors, the logistic regression analysis showed that those aged 60–69 were 39% less likely to have behavioral deviations as compared with those respondents aged 35–59. For enabling factors, compared with the patients who spent less than 3,000 CNY on healthcare last year, those who reported their previous year’s healthcare expenses between 3,000 and 4,999 CNY (aOR = 4.89, 95%CI = 2.78–8.61), between 5,000 and 9,999 CNY (aOR = 6.84, 95%CI = 3.84–12.17) and greater than 10,000 CNY (aOR = 19.02, 95%CI = 8.23–43.99) were more likely to change their original willingness and seek care from non-PHC institutions. Compared to those covered by UEBMI, those who were covered by URRBMI (aOR = 0.30, 95%CI = 0.13–0.70) were less likely to have behavioral deviations. Compared with the patients who did not apply for medical reimbursement, those who considered the medical cost reimbursement very convenient (aOR = 0.38, 95%CI = 0.22–0.65) were also less likely to have behavioral deviations. For the need factors, compared to people who did not visit PHC institutions in the previous year, individuals with PHC visits due to illness last year were 61% less likely to have behavioral deviations.

Among the group of patients with multiple chronic diseases, for predisposing factors, compared to those aged 35–59, respondents aged 70–75 (aOR = 0.34, 95%CI = 0.16–0.73) were less likely to have behavioral deviations. For enabling factors, when compared with those having the least healthcare expenditures last year, those who reported their previous year’s healthcare expenses between 5,000 and 9,999 CNY (aOR = 4.20, 95%CI = 2.08–8.50) or greater than 10,000 CNY (aOR = 14.19, 95%CI = 7.14–28.22) were more likely to change their original willingness and seek care from non-PHC institutions. Moreover, compared with the patients who didn’t apply for medical reimbursement, those who considered the medical cost reimbursement convenient (aOR = 0.27, 95%CI = 0.11–0.70) or very convenient (aOR = 0.28, 95%CI = 0.13–0.62) were less likely to have behavioral deviations. For need factors, the patients with PHC visits due to illness last year (aOR = 0.28, 95%CI = 0.16–0.48) were also less likely to change their original willingness and seek care from non-PHC institutions than those who did not visit PHC institutions in the previous year. Besides, patients with polypharmacy situations were 42% less likely to have behavioral deviations than those without (aOR = 0.58, 95%CI = 0.34–0.97).

## Discussion

To our knowledge, there were only few studies to investigate behavioral deviations among chronic disease patients who were willing to visit PHC institutions and their determinants. Results from this study demonstrated that approximately two in five chronic disease patients changed their original intentions and actually visited non-PHC institutions. Predisposing factor (age), enabling factors (healthcare expenditure in the previous year, type of medical insurance and convenience of medical cost reimbursement), and need factors (PHC visits due to illness last year and polypharmacy situations) were significant predictors of behavioral deviations among chronic disease patients. This finding suggested that even under the context of the rising prevalence of non-communicable disease, the increase in seeking health services from PHC institutions was still limited, despite of the high demand of PHC among chronic disease patients [43]. Therefore, it is important to explore chronic disease patients’ healthcare-seeking behavior, especially those who were originally willing to visit PHC institutions. Previous studies have shown that it is crucial for forming a rational care process and improving the efficiency of the national healthcare system [44, 45].

### Predisposing factors

The present study found that older patients with chronic diseases were more likely to maintain their willingness to visit PHC institutions, which supports the findings reported by Nicholas [46]. This could be attributed to the fact that, when compared to younger patients with chronic diseases, older patients have limited mobility and may experience a more significant decline in their physiological functions [42]. Therefore, the elderly are more likely to choose PHC institutions with better geographical accessibility when disease occurs. Furthermore, financial constraints are a long-term consideration for these seniors [45]. PHC institutions can provide affordable chronic disease management, and this may attract seniors to maintain their willingness to visit PHC institutions.

### Enabling factors

Previous studies have shown that need factors were strong determinants of healthcare-seeking behavior in older adults and had greater predictive value compared to the predisposing and enabling variables in Andersen’s model [45]. Interestingly, we found that the enabling factors were strongly significant factors associated with chronic disease patients’ healthcare-seeking behavior. Previous studies have shown that older patients with chronic diseases have greater healthcare expenditure than those without chronic diseases [28], so economic and social factors have a greater impact on their health service utilization behavior. In the present study, the healthcare expenditure in the previous year was the most significant factor associated with behavioral deviations among chronic disease patients who were willing to visit PHC institutions. And our study found that those who spent more on healthcare last year tended to change their original willingness and seek care from non-PHC institutions. This may be attributed to chronic disease patients’ reluctance to give up medical costs invested in the previous year. Consequently, they were more likely to pursue aggressive and effective treatment [47]. On the other hand, this was probably due to the distrust of some chronic disease patients in the technical capacity of PHC institutions [48]. Thus, it is recommended to introduce highly qualified personnel and provide professional training to the current PHC workforce to improve the capability of PHC institutions.

Besides, chronic disease patients who considered medical reimbursement convenient, as well as those covered by the URRBMI, were more likely to maintain their original willingness and actually visited PHC institutions. Previous studies showed that the reimbursement of health insurance affected patients’ health services utilization [39]. In line with previous studies, we also found that the convenience of medical cost reimbursement largely promoted patients’ primary health service utilization. This suggests that establishing well-developed health insurance reimbursement schemes would benefit promoting patients’ choice of PHC institutions. In addition, the likelihood of behavioral deviations was higher among chronic disease patients covered by the UEBMI. The main reason for this difference is that chronic disease patients covered by UEBMI usually had a more stable source of income than those covered by URRBMI, thus they could afford higher medical costs and pursued aggressive treatment.

### Need factors

Other influencing factors of behavioral deviations among chronic disease patients with a willingness to visit PHC institutions included PHC visits due to illness and polypharmacy situations. We speculated that as the majority of chronic disease patients taking multiple medications were elderly, travel to non-PHC institutions was more costly and difficult for older adults [49]. Therefore, chronic disease patients who took multiple medications were 45% less likely to have behavioral deviations than those without. Consistent with the conclusions reported by many previous studies [50, 51], our study also indicated that personal medical visit experience is a core criterion in patients’ subsequent decisions for or against hospitals. The result showed that those who visited PHC institutions due to illness last year were 65% less likely to have behavioral deviations than those who did not visit PHC institutions in the previous year. This may be due to the fact that some patients may increase their trust as they witnessed the technical capacity improvement in PHC institutions during previous primary care experiences [52]. Moreover, chronic disease patients may also experience the benefits of lower costs and better transportation availability [45], so they were willing to obtain care from PHC institutions. More efforts are required in PHC institutions to improve continuity and individualization of care in order to increase patient-provider trust.

Importantly, the differences in factors that affected behavioral deviations from the original intention to visit PHC institutions between single chronic disease patients and multiple chronic disease patients were also noteworthy in our study. Results from this study found that the single chronic disease patients covered by URRBMI were less likely to have behavioral deviations. Interestingly, however, medical insurance coverage for patients with multiple chronic diseases was not associated with their healthcare-seeking behavior. The findings suggested that the degree of financial compensation did not affect the healthcare-seeking behavior of the patients with multimorbidity, which was consistent with the previous study [4]. The plausible reason may be that, as compared to patients with a single chronic disease, the patients with multimorbidity have poorer physiological function and more psychological distress [53], which may cause them to prioritize the technical capacity of hospitals rather than financial compensation. In addition, although multimorbidity patients taking multiple medications tended to maintain their original willingness and visit PHC institutions, a similar result was not noted among patients with a single chronic disease. It may be because multimorbidity patients taking multiple medications had a long history of the disease. Hence, they had a deeper understanding of their conditions and would choose to visit PHC institutions with convenient transportation when their disease was relatively stable [53].

Based on the research findings, several intervention strategies can be highlighted to further reduce behavioral deviations and promote PHC institution visits among chronic disease patients. First, the health insurance reimbursement system for chronic diseases should be improved. In particular, health insurance coverage and the reimbursement rate for multimorbidity patients must be improved to reduce patients’ financial burden and to ensure better access to health services. Second, PHC institutions ought to deliver comprehensive, person-centered care as well as focus on continuity and individualized care to improve the utilization of PHC institutions in China. Third, changing the public perceptions of low-quality care in PHC institutions, particularly among multimorbidity patient groups, is essential to encourage reasonable healthcare-seeking behavior, as well as to improve the efficiency of the overall healthcare system.

In comparison with previous studies, this study is strengthened by three features [15–17, 44, 45]. First, this study focuses on chronic disease patients who were originally willing to visit PHC institutions. From the view of the research subjects, this group is much easier to implement the intervention and achieve the desired effect of forming a rational flow of care. Second, this study used Andersen’s behavioral model as the theoretical framework, which could provide a basis for systematic investigations of factors influencing healthcare-seeking behavior. Third, this study compared the reasons for deviations in healthcare-seeking behavior between single chronic disease patients and multiple chronic disease patients, which deepened the research content. However, there are still some limitations that should be mentioned. First, the cross-sectional design of this study has its limitation in determining the causality relationship between the healthcare-seeking behavior and the potential influencing factors. Second, there is potential recall bias or desirability bias since the survey data were based on self-reported. Third, due to time and fund constraints, the potential influencing factors on behavioral deviations included in this study may be incomplete. Fourth, since the study was based on the Andersen model, the characteristics of PHC that may be actionable, as well as some interactions between patients and the health system, might be ignored. In addition, the influence of the low response rate and the COVID-19 epidemic on sample representativeness may be a concern. Thus, it is strongly recommended for future studies to repeat data collection at different time points to form longitudinal data to enable causal inference. Also, some other potential influencing factors can be further included, such as accessibility of health care services, self-rated health, etc.

## Conclusion

This study described behavioral deviations among chronic disease patients with original intention to visit PHC institutions and identified its influencing factors. To the best of our knowledge, this is the first study to investigate behavioral deviations and their related factors respectively for a group of patients with single and multiple chronic diseases, which is expected to shed light on future research in this area. The results suggested that a number of predisposing factors (age), enabling factors (healthcare expenditure in the previous year and convenience of medical cost reimbursement), and need factors (PHC visits due to illness last year) were found to be the common influencing factors of behavioral deviations for both the group of patients with single and multiple chronic diseases. Therefore, measures to promote chronic disease patients’ access to PHC institutions should focus on implementing a more advanced health insurance system, strengthening the technical capacity of PHC institutions and steadily developing a new concept of orderly healthcare-seeking behavior among chronic disease patients.

## Supplementary Information

**Additional file 1.**

**Additional file 2.**

**Additional file 3.**

**Additional file 4: Table A1.** Robustness test (supplementary variables). **Table A2. **Robustness test (replace explanatory variables).

## Data Availability

The datasets generated during and/or analyzed during the current study are available from the corresponding author on reasonable request.

## Abbreviations

PHC: Primary health care
UEBMI: Urban Employee Basic Medical Insurance
URRBMI: Urban-Rural Resident Basic Medical Insurance
CNY: Chinese Yuan
uOR: Unadjusted Odds Ratio
aOR: Adjusted Odds Ratio
CI: Confidence interval
