# Supplementary material for: Behavioral deviations: healthcare-seeking behavior of chronic disease patients with intention to visit primary health care institutions
Source: BMC Health Serv Res. 2023 May 16;23:490. doi: 10.1186/s12913-023-09528-y (PMC10185376; doi:10.1186/s12913-023-09528-y)
Supplement: Supplementary file 1 — Additional file 1. [file 12913_2023_9528_MOESM1_ESM.doc]

**Questionnaire for a prospective cohort of**

**residents with chronic disease in Fuqing City**

**Respondent’s information**

1. Name:
2. ID number:
3. Telephone number:
4. Address

County:

Town:

Village:

**Part 1. Personal Basic Information**

| Question  number | Questions and options | Answer |
| --- | --- | --- |
| 1 | Record of the respondent’s sex  1) Male  2) Female |  |
| 2 | How old are you?  1) 35-59 years old  2) 60-69 years old  3) 70-75 years old |  |
| 3 | What is your highest educational attainment?  1) Illiteracy  2) Elementary school  3) Middle school  4) High school or higher |  |
| 4 | What is your marital status?  1) Others (Single,divorced,widowed and separated)  2) Married |  |

**Part 2. Personal Health Condition**

| Question  number | Questions and options | Answer |
| --- | --- | --- |
| 5 | Have you ever been diagnosed with diseases by doctors at township/district hospitals or above?  1) Yes  2) No  *Notes: If the respondent answered “No”, skip to question 6.* |  |
| 5.1 | Do you have diabetes?  1) Yes  2) No |  |
| 5.2 | Do you have coronary heart disease?  1) Yes  2) No |  |
| 5.3 | Do you have a stroke?  1) Yes  2) No |  |
| 5.4 | Do you have hypertension?  1) Yes  2) No |  |
| 5.5 | Do you have hyperuricemia?  1) Yes  2) No |  |
| 5.6 | Do you have cancer?  1) Yes  2) No |  |
| 5.7 | Do you have liver disease?  1) Yes  2) No |  |
| 5.8 | Do you have hyperthyroidism?  1) Yes  2) No |  |
| 5.9 | Do you have hypothyroidism?  1) Yes  2) No |  |
| 5.10 | Do you have thyroid nodules?  1) Yes  2) No |  |
| 6 | Are you taking two or more classes of drugs (polypharmacy)?  1) Yes  2) No |  |

**Part 3. Health Service Demand**

| Question  number | Questions and options | Answer |
| --- | --- | --- |
| 7 | Which healthcare provider did you prefer to visit when you had common or frequently-occurring diseases?   1. Primary health care institutions (Village clinic, community healthcare center, and township hospital) 2. Non-primary health care institutions (General hospital,   Chinese medicine hospital, and specialized hospital) |  |
| 8 | In the past year, which type of medical institutions did you visit when you had common or frequently-occurring diseases?  1) Primary health care institutions (Village clinic, community healthcare center, and township hospital)  2) Non-primary health care institutions (General hospital,  Chinese medicine hospital, and specialized hospital)  *Notes: If the respondent answered “No”, skip to question 9.* |  |
| 9 | Have you visited primary health care institutions due to common diseases and frequently-occurring diseases in the previous year?  1) Yes  2) No |  |
| 10 | What is your annual household income?   1. 0-29,999 Chinese Yuan (CNY) 2. 30,000-99,999 CNY 3. 100,000-149,999 CNY 4. 150,000 CNY and above |  |
| 11 | In the past year, the total cost of your health care expenditures was?  1) 0-2,999 CNY  2) 3,000-4,999 CNY  3) 4,999-9,999 CNY  4) 1,0000 CNY and above |  |
| 12 | What kind of medical insurance cover you?  1) Self-pay  2) Urban Employee Basic Medical Insurance (UEBMI)  3) Urban-Rural Resident Basic Medical Insurance (URRBMI) |  |
| 13 | Do you think medical cost reimbursement is convenient at the medical institution you often visit?  1) Not reimbursed  2) Inconvenient  3) Convenient  4) Very convenient |  |
| 14 | Do you need guidance on your health issues?   1. Yes 2. No |  |
