# Supplementary material for: Behavioral deviations: healthcare-seeking behavior of chronic disease patients with intention to visit primary health care institutions
Source: BMC Health Serv Res. 2023 May 16;23:490. doi: 10.1186/s12913-023-09528-y (PMC10185376; doi:10.1186/s12913-023-09528-y)
Supplement: Supplementary file 2 — Additional file 2. [file 12913_2023_9528_MOESM2_ESM.doc]

**Participant selection**
