# Supplementary material for: Behavioral deviations: healthcare-seeking behavior of chronic disease patients with intention to visit primary health care institutions
Source: BMC Health Serv Res. 2023 May 16;23:490. doi: 10.1186/s12913-023-09528-y (PMC10185376; doi:10.1186/s12913-023-09528-y)
Supplement: Supplementary file 3 — Additional file 3. [file 12913_2023_9528_MOESM3_ESM.doc]

Multiple collinearity test between different analyzed factors

| Variables | Total | |  | Single chronic disease | |  | Multimorbidity | |
| --- | --- | --- | --- | --- | --- | --- | --- | --- |
|  | Tolerance | VIF |  | Tolerance | VIF |  | Tolerance | VIF |
| Gender | 0.680 | 1.470 |  | 0.679 | 1.472 |  | 0.642 | 1.557 |
| Age | 0.806 | 1.241 |  | 0.769 | 1.300 |  | 0.861 | 1.161 |
| Education | 0.670 | 1.493 |  | 0.700 | 1.429 |  | 0.606 | 1.650 |
| Marital status | 0.953 | 1.050 |  | 0.938 | 1.066 |  | 0.957 | 1.045 |
| Health care expenditure in the previous year | 0.938 | 1.067 |  | 0.958 | 1.043 |  | 0.944 | 1.059 |
| Medical insurance | 0.966 | 1.035 |  | 0.970 | 1.031 |  | 0.939 | 1.065 |
| Convenience of medical cost reimbursement | 0.995 | 1.005 |  | 0.998 | 1.003 |  | 0.978 | 1.022 |
| Primary care experience | 0.980 | 1.020 |  | 0.982 | 1.018 |  | 0.962 | 1.039 |
| Polypharmacy | 0.886 | 1.128 |  | 0.931 | 1.074 |  | 0.893 | 1.119 |
| Need of guidance on health issues | 0.984 | 1.016 |  | 0.976 | 1.025 |  | 0.989 | 1.011 |
