# Supplementary material for: Behavioral deviations: healthcare-seeking behavior of chronic disease patients with intention to visit primary health care institutions
Source: BMC Health Serv Res. 2023 May 16;23:490. doi: 10.1186/s12913-023-09528-y (PMC10185376; doi:10.1186/s12913-023-09528-y)
Supplement: Supplementary file 4 — Additional file 4: Table A1. Robustness test (supplementary variables). Table A2. Robustness test (replace explanatory variables). [file 12913_2023_9528_MOESM4_ESM.doc]

**Table A1.** Robustness test (supplementary variables)

| Variables | Total |  | Single chronic disease |  | Multimorbidity |
| --- | --- | --- | --- | --- | --- |
|  | *aOR* (95%*CI*) |  | *aOR* (95%*CI*) |  | *aOR* (95%*CI*) |
| **Predisposing factors** |  |  |  |  |  |
| Sex (Ref=Male) |  |  |  |  |  |
| Female | 0.87 (0.60-1.26) |  | 0.95 (0.59-1.54) |  | 0.71 (0.37-1.36) |
| Age (Ref=35-59) |  |  |  |  |  |
| 60-69 | 0.58 (0.41-0.82)** |  | 0.60 (0.38-0.95)* |  | 0.57 (0.31-1.01) |
| 70-75 | 0.46 (0.28-0.74)** |  | 0.55 (0.29-1.05) |  | 0.32 (0.15-0.70)** |
| Education (Ref=Illiteracy) |  |  |  |  |  |
| Elementary school | 1.12 (0.78-1.61) |  | 0.97 (0.61-1.55) |  | 1.52 (0.82-2.82) |
| Middle school | 1.09 (0.69-1.72) |  | 0.78 (0.44-1.39) |  | 2.08 (0.91-4.75) |
| High school or higher | 1.29 (0.68-2.47) |  | 1.25 (0.57-2.76) |  | 1.44 (0.43-4.82) |
| Marital status (Ref=Others) | |  |  |  |  |
| Married | 0.65 (0.40-1.06) |  | 0.62 (0.32-1.21) |  | 0.67 (0.32-1.38) |
| **Enabling factors** |  |  |  |  |  |
| Health care expenditure  in the previous year (Ref=<3,000) | |  |  |  |  |
| 3,000-4,999 | 3.38 (2.17-5.27)*** |  | 4.93 (2.78-8,72)*** |  | 1.79 (0.82-3.90) |
| 5,000-9,999 | 5.80 (3.75-8.99)*** |  | 7.19 (4.02-12.87)** |  | 4.18 (2.04-8.44)*** |
| >=10,000 | 15.38 (9.35-25.30)*** |  | 20.06 (8.58-46.86)*** |  | 14.10 (7.06-28.13)*** |
| Medical insurance (Ref=UEBMI) | |  |  |  |  |
| URRBMI | 0.31 (0.13-0.72)** |  | 0.25 (0.08-0.80)* |  | 0.42 (0.11-1.58) |
| Convenience of medical cost reimbursement  (Ref=No reimbursed) | |  |  |  |  |
| Inconvenient | 0.68 (0.37-1.25) |  | 0.72 (0.32-1.60) |  | 0.62 (0.23-1.69) |
| Convenient | 0.50 (0.31-0.82)** |  | 0.63 (0.34-1.14) |  | 0.26 (0.10-0.67)** |
| Very convenient | 0.36 (0.23-0.55)*** |  | 0.39 (0.23-0.67)** |  | 0.27 (0.12-0.60)** |
| Annual household income (Ref=<30,000) | |  |  |  |  |
| 30,000-99,999 | 0.78 (0.53-1.13) |  | 0.83 (0.52-1.33) |  | 0.58 (0.28-1.19) |
| 100,000-149,999 | 1.09 (0.73-1.63) |  | 1.35 (0.81-2.28) |  | 0.80 (0.42-1.54) |
| >=150,000 | 1.06 (0.62-1.82) |  | 0.92 (0.46-1.83) |  | 1.52 (0.61-3.77) |
| **Need factors** |  |  |  |  |  |
| Primary care experience (Ref=No) | |  |  |  |  |
| Yes | 0.35 (0.25-0.47)*** |  | 0.38 (0.26-0.57)*** |  | 0.27 (0.16-0.48)*** |
| Polypharmacy (Ref=No) |  |  |  |  |  |
| Yes | 0.53 (0.37-0.76)** |  | 0.62 (0.36-1.09) |  | 0.57 (0.34-0.96)* |
| Need of guidance on health issues (Ref=No) | |  |  |  |  |
| Yes | 0.85 (0.63-1.17) |  | 0.91 (0.61-1.36) |  | 0.73 (0.44-1.23) |

**P* < 0.05, ***P* < 0.01, ****P* < 0.001. aOR = adjusted odds ratio.

**Table A2.** Robustness test (replace explanatory variables)

| Variables | Total |  | Single chronic disease |  | Multimorbidity |
| --- | --- | --- | --- | --- | --- |
|  | *aOR* (95%*CI*) |  | *aOR* (95%*CI*) |  | *aOR* (95%*CI*) |
| **Predisposing factors** |  |  |  |  |  |
| Sex (Ref=Male) |  |  |  |  |  |
| Female | 0.86 (0.60-1.25) |  | 0.98 (0.61-1.58) |  | 0.70 (0.37-1.32) |
| Age (Ref=35-59) |  |  |  |  |  |
| 60-69 | 0.60 (0.42-0.84)** |  | 0.61 (0.39-0.96)* |  | 0.59 (0.33-1.03) |
| 70-75 | 0.47 (0.29-0.76)** |  | 0.53 (0.28-1.00) |  | 0.34 (0.16-0.73)** |
| Education (Ref=Illiteracy) |  |  |  |  |  |
| Elementary school | 1.11 (0.77-1.59) |  | 0.97 (0.61-1.56) |  | 1.39 (0.76-2.54) |
| Middle school | 1.07 (0.67-1.68) |  | 0.78 (0.44-1.39) |  | 1.92 (0.85-4.33) |
| High school or higher | 1.29 (0.68-2.46) |  | 1.30 (0.59-2.84) |  | 1.32 (0.39-4.43) |
| Marital status (Ref=Others) | |  |  |  |  |
| Married | 0.66 (0.41-1.08) |  | 0.61 (0.31-1.20) |  | 0.70 (0.34-1.45) |
| **Enabling factors** |  |  |  |  |  |
| Health care expenditure  in the previous year (Ref=<3,000) | |  |  |  |  |
| 3,000-4,999 | 3.42 (2.20-5.32)*** |  | 4.98 (2.83-8.78)*** |  | 1.85 (0.86-3.99) |
| 5,000-9,999 | 5.72 (3.69-8.85)*** |  | 6.95 (3.90-12.41)*** |  | 4.23 (2.09-8.57)*** |
| >=10,000 | 15.33 (0.32-25.21)*** |  | 19.48 (8.40-45.16)*** |  | 14.06 (7.03-28.15)*** |
| Medical insurance (Ref=UEBMI) | |  |  |  |  |
| URRBMI | 0.30 (0.13-0.70)** |  | 0.25 (0.08-0.79)* |  | 0.38 (0.10-1.49) |
| Convenience of medical cost reimbursement  (Ref=No reimbursed) | |  |  |  |  |
| Inconvenient | 0.68 (0.37-1.25) |  | 0.70 (0.31-1.55) |  | 0.69 (0.26-1.87) |
| Convenient | 0.50 (0.31-0.82)** |  | 0.63 (0.35- 1.13) |  | 0.28 (0.11-0.71)** |
| Very convenient | 0.35 (0.23-0.55)*** |  | 0.38 (0.22-0.64)*** |  | 0.28 (0.13-0.61)** |
| **Need factors** |  |  |  |  |  |
| Primary care experience (Ref=No) | |  |  |  |  |
| Yes | 0.35 (0.25-0.47)*** |  | 0.39 (0.26-0.58)*** |  | 0.27 (0.16-0.47)*** |
| Categories of medication taken (Ref=1 category) | |  |  |  |  |
| 2-3 categories | 0.50 (0.34-0.73)*** |  | 0.63 (0.36-1.10) |  | 0.49 (0.28-0.86)* |
| 4 or more categories | 0.95 (0.44-2.05) |  | 4.58 (0.24-86.919) |  | 1.10 (0.46-2.61) |
| Need of guidance on health issues (Ref=No) | |  |  |  |  |
| Yes | 0.85 (0.62-1.16) |  | 0.91 (0.61-1.36) |  | 0.72 (0.43-1.21) |

**P* < 0.05, ***P* < 0.01, ****P* < 0.001. aOR = adjusted odds ratio.
